# Lymphoepithelial cyst of the pancreas: A case report and summary of imaging features of pancreatic cysts

**DOI:** 10.1016/j.ijscr.2019.01.022

**Published:** 2019-01-30

**Authors:** Yosuke Namba, Akihiko Oshita, Takashi Nishisaka, Maiko Namba, Tamito Sasaki, Yasuhiro Matsugu, Toshiyuki Itamoto

**Affiliations:** a Department of Gastroenterological Surgery, Hiroshima Prefectural Hospital, Japan; b Department of Gastroenterological and Transplant Surgery, Applied Life Sciences, Institute of Biomedical & Health Sciences, Hiroshima University, Japan; c Department of Pathology Clinical Laboratory, Hiroshima Prefectural Hospital, Japan; d Department of Gastroenterology, Hiroshima Prefectural Hospital, Japan

**Keywords:** LEC, lymphoepithelial cyst, MRI, magnetic resonance imaging, MCN, mucinous cystic neoplasm, IPMN, intraductal papillary mucinous neoplasm, CT, computed tomography, CEA, carcinoembryonic antigen, CA19-9, carbohydrate antigen 19-9, EUS, endoscopic ultrasonography, ERCP, endoscopic retrograde cholangiopancreatography, Lymphoepithelial cyst, Imaging, Features, Pancreatic, Cyst

## Abstract

**Introduction:**

A lymphoepithelial cyst (LEC) of the pancreas is a benign and rare lesion that is difficult to diagnose preoperatively based on imaging studies.

**Presentation of case:**

We report a case of a 49-year-old man who presented with weight loss and diarrhea. The serum carcinoembryonic antigen and carbohydrate antigen 19-9 levels were slightly elevated to 6.7 ng/mL (reference value <5.0 ng/mL) and 45 U/mL (reference value <37 U/mL), respectively. Computed tomography showed a large cystic mass with internal septa in the pancreatic tail. The cystic wall and the septa showed enhancement while the cystic contents remained unenhanced. Magnetic resonance imaging (MRI) demonstrated a multiple-ball-like lesion with low signal intensity on T1-weighted image and high signal intensity on T2-weighted image. Diffusion-weighted MRI showed high signal intensity in the central and iso-signal intensity in the peripheral portions of the cystic lesion. The cystic wall and septa showed high signal intensity, and the cystic contents showed low signal intensity on an enhanced MRI. Endoscopic ultrasonography showed a cyst with multiple high-echoic lesions in the pancreatic tail. A mucinous cystic neoplasm and branch duct intraductal papillary mucinous neoplasm were considered among the differential diagnoses, and we performed distal pancreatectomy with concomitant splenectomy and lymphadenectomy for both diagnostic and therapeutic purposes. Histopathological findings revealed that the cystic wall was lined by stratified squamous epithelium, and several lymphoid follicles and a few sebaceous glands were observed within the cystic wall without hair follicles, leading to the final diagnosis of an LEC.

**Conclusion:**

We report a rare case of a pancreatic LEC, which was difficult to be diagnosed and summarize the imaging features of pancreatic cysts to differentiate from the malignancy preoperatively.

## Highlights

• A lymphoepithelial cyst (LEC) of the pancreas is a benign and rare lesion.
• A pancreatic LEC is difficult to be diagnosed and differentiate from the malignancy preoperatively.
• We summarized the imaging features of pancreatic cysts to differentiate from the malignancy.

## 1 Introduction

A pancreatic lymphoepithelial cyst (LEC) is a rare and benign pancreatic lesion. Pancreatic LECs were first described by Luchtrath and Schriefers in 1985 [1]. Pancreatic LECs are usually observed in middle-aged and elderly men and occur with equal frequency in the pancreatic head, body, or tail and may present as a uni- or multilocular lesion [[2], [3], [4]]. Pancreatic LECs are indistinguishable from other pancreatic lesions including serous cystic neoplasm and mucinous cystic neoplasm (MCN), intraductal papillary mucinous neoplasm (IPMN), as well as dermoid cyst and epidermoid cyst because preoperative imaging studies show marked variations in the presentation of LECs among patients, and these often mimic other pancreatic lesions [2]. We report a case of a pancreatic LEC and summarize the imaging features of pancreatic cystic lesions. This work has been reported in line with the SCARE criteria [5].

## 2 Presentation of case

**Fig. 1:**
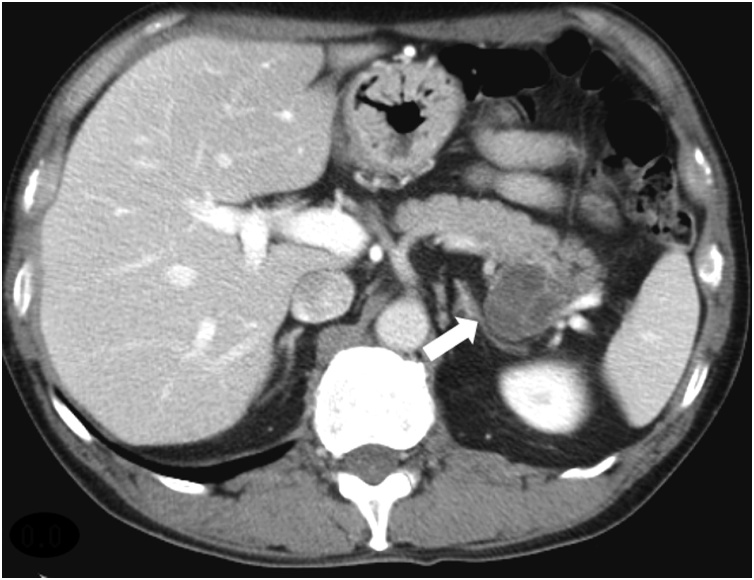
Computed tomography (CT): Enhanced CT showed enhancement of the cystic wall and septa while the cystic contents appeared unenhanced (arrow).

**Fig. 2:**
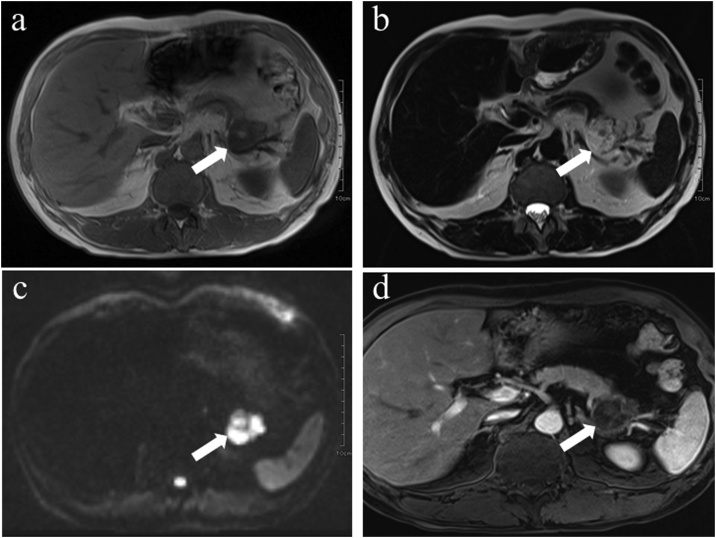
Magnetic resonance imaging (MRI): A. MRI demonstrated a multiple-ball-like lesion of low signal intensity on T1-weighted image (arrow). B. MRI showed the same lesion with high signal intensity on T2-weighted image (arrow). C. MRI showed high signal intensity in the central and iso-signal intensity in the peripheral portions of the cystic lesion on diffusion-weighted image (arrow). D. Enhanced MRI showed high signal intensity in the cystic wall and septa and low signal intensity of the cystic contents (arrow).

A 49-year-old man complained of diarrhea and weight loss. He received 3 mg of prednisolone per day for the treatment of the diagnosed polymyositis. Computed tomography (CT) revealed a multilocular cyst in the pancreatic tail, and he was referred to our hospital for the further examination. Laboratory data showed that complete blood cell count, and hepatic and renal functions were within the normal limits. The c-reactive protein level was within the normal limits owing to the steroid therapy. The serum levels of carcinoembryonic antigen (CEA) and carbohydrate antigen 19-9 (CA19-9) were slightly elevated to 6.7 ng/mL (reference value <5.0 ng/mL) and 45 U/mL (reference value <37 U/mL), respectively. Enhanced abdominal CT showed a large cystic mass with internal septa in the pancreatic tail. The cystic wall and the septa showed enhancement; however, the cystic contents remained unenhanced (Fig. 1). Magnetic resonance imaging (MRI) demonstrated a multiple-ball-like lesion with low signal intensity on T1-weighted image and high signal intensity on T2-weighted image (Fig. 2a, b). Diffusion-weighted MRI showed high signal intensity in the central portion and iso-signal intensity in the peripheral portion of the cystic lesion (Fig. 2c). The cystic wall and the septa showed high signal intensity, and the cystic contents showed low signal intensity on an enhanced MRI (Fig. 2d).

**Fig. 3:**
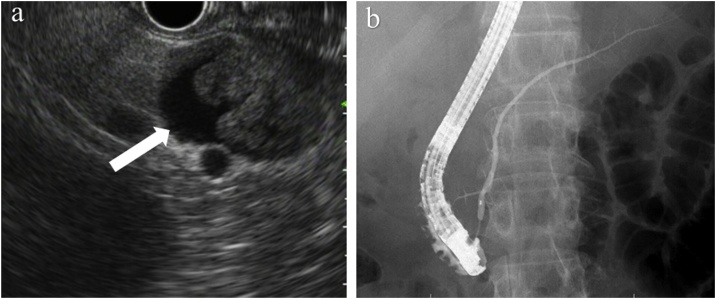
Endoscopic imaging: A. Endoscopic ultrasonography (EUS) showed a cyst with multiple high-echoic lesions in the pancreatic tail (arrow). B. Endoscopic retrograde cholangiopancreatographic image showed a normal main pancreatic duct and no communication between the main pancreatic duct and the cystic lesion.

**Fig. 4:**
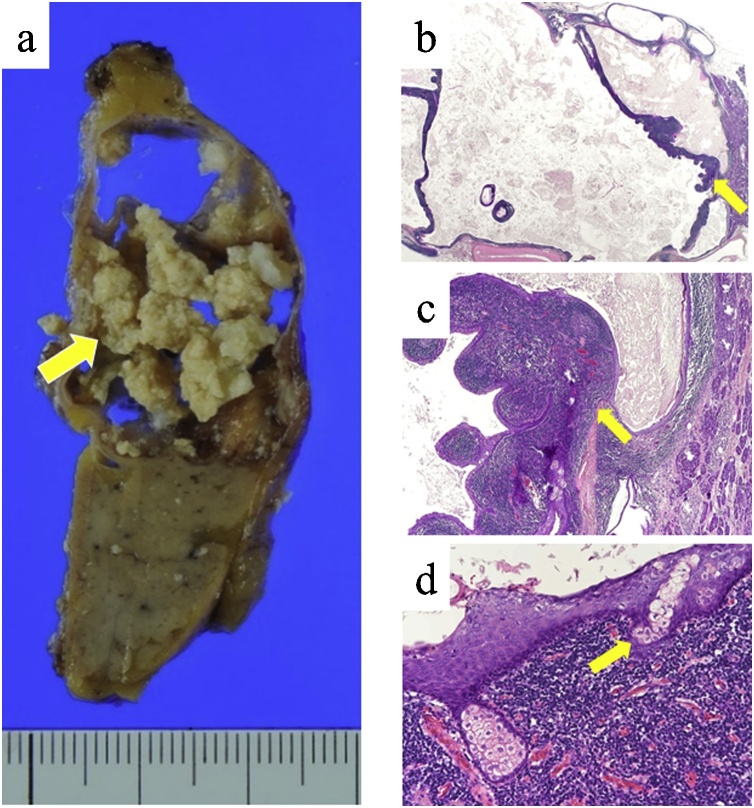
Histopathology: A. The cut surface of the resected specimen showed a multilocular cyst with solid nodules (arrow). B. The cyst was separated by a band of lymphoid tissue (arrow) (hematoxylin & eosin [H&E] stain ×10). C. The cystic wall was primarily lined by stratified squamous epithelium, and the cyst contains keratinized material. Several subepithelial lymphoid follicles could be observed (arrow) (H&E stain ×40). D. Sebaceous glands could be observed within the stratified squamous epithelium (arrow) (H&E stain ×400).

Endoscopic ultrasonography (EUS) showed a cyst with multiple high-echoic lesions in the pancreatic tail (Fig. 3a). Endoscopic retrograde cholangiopancreatography (ERCP) showed a normal main pancreatic duct and no communication between the main pancreatic duct and the cystic lesion (Fig. 3b). MCN and IPMN were considered among the preoperative differential diagnoses. The nonspecific view for MCN was that the patient was male and that for IPMN was no communication between the main pancreatic duct and the cystic lesion. We performed distal pancreatectomy with concomitant splenectomy and lymphadenectomy for both diagnostic and therapeutic purposes. The operative duration was 217 min, and the bleeding amount was 446 mL. Postoperative course was uneventful. The cut surface of the resected specimen revealed a multilocular cyst with solid nodules (Fig. 4a). Histopathological findings revealed that the cystic wall was lined by stratified squamous epithelium, and several lymphoid follicles and a few sebaceous glands were located in the cystic wall without hair follicles, leading to the final diagnosis of a pancreatic LEC (Fig. 4b–d). The diarrhea and weight loss were cured 3 years after the surgery.

## 3 Discussion

Pancreatic LECs are extremely rare. It is reported to be approximately 0.5% of pancreatic cysts [2]. Luchtrath et al. ﬁrst reported LECs in 1985 [1], and Truong et al. named these lesions lymphoepithelial cysts of the pancreas in 1987 [6]. Mege et al. reported a study in which pancreatic LECs were usually observed in middle-aged to elderly men (mean age 55 years, range 20–82 years, 91 men vs. 26 women) and also observed that this lesion was occasionally accompanied by abdominal pain (43%) and an elevated serum CA 19-9 level (55%) [3]. Pancreatic cysts can be classified into true cysts, pseudocysts, and cystic neoplasms [7,8]. LEC is a type of true cyst characterized by a lining of squamous epithelium with dense subepithelial lymphoid tissue. The cystic contents are usually white in color and may include keratinized material or cholesterol crystals [[9], [10], [11]].

Preoperative diagnosis of pancreatic LECs and differentiation with the malignancy are difficult. Serum levels of CEA, CA19-9, CA-125, cancer-related antigen 72-4, and mucin-like carcinoma-associated antigen, and ﬂuid viscosity are expected to be signiﬁcantly lower in patients with LECs than in those with mucinous neoplasms [9,12,13]. A few patients have shown high levels of CA19-9 in the cystic fluid (not in the serum) [14]. LECs contain keratinized debris resulting in a typical caseous appearance [15]. CT demonstrated enhancement of the cystic wall and the septa in this patient. The cyst itself showed low density uniform without enhancement [9]. The cystic contents of the LEC included keratin; thus, MRI showed higher signal intensity than that of free water on T1-weighted image and lower signal intensity on T2-weighted image and higher signal intensity on diffusion-weighted image [15,16]. Based on EUS evaluation, LECs might vary between simple round cysts and multiloculated complex cystic lesions, depending upon the degree of keratin formation [14,17]. Pancreatic LECs are usually round with a well-deﬁned wall that is sharply demarcated from the pancreas and the surrounding adipose tissue [9].

In order to differentiate from the malignancy preoperatively, we summarized the imaging features of pancreatic cysts that require the surgical treatment (Table 1). In our patient, the findings observed on enhanced CT, diffusion-weighted MRI, and EUS were consistent with the features of an LEC, whereas the MRI findings on T1-wighted image and T2-weighted image were not consistent with those of LEC. To sum up these findings, We performed a distal pancreatectomy with concomitant splenectomy and lymphadenectomy as we could not rule out MCN and IPMN.

**Table 1:** Imaging features of pancreatic cysts.

	Location	CT	MRI	EUS
LEC Dermoid cyst Epidermoid cyst	Any	Enhancing wall and septum Low density cystic lesion without enhancement	Higher intensity than water on T1WI (depending on the keratin) Lower intensity than water on T2WI Higher intensity than water on DWI (depending on the keratin)	Mosaic pattern (depending on the keratin)
IPMN	Any	Multilocular Enhancing nodule	Well circumscribed	Lobular Multilocular Multiple cysts Pancreatic duct diameter >5mm Communication with pancreatic duct
MCN	Body Tail	Enhancing capsule Cyst in cyst, Mural cyst Enhancing mural nodule Calcification of mural nodule	Low intensity on T1WI (bleeding: high intensity) High intensity on T2WI	Smooth unilocular Peripheral calcification Thick wall
SCN	Any	Clustered of microcyst Enhancement on arterial-phase (Solid type)	Low intensity on T1WI (bleeding: high intensity) High intensity, low septum on T2WI	Lobular Multilocular Well circumscribed Acoustic enhancement

LEC; lymphoepithelial cyst, IPMN; intraductal papillary mucinous neoplasm, MCN; mucinous cystic neoplasm,SCN; serous cystic neoplasm, CT; computed tomography, MRI; magnetic resonance imaging, EUS; endoscopic ultrasonography.

Although LECs are difficult to be diagnosed preoperatively based on imaging studies, it can be easily distinguished by its characteristic histomorphological features. Microscopically, LECs are characterized by cysts lined by stratiﬁed squamous epithelium with adjacent dense subepithelial lymphoid tissue containing lymphoid follicles [2]. In terms of microscopic differential diagnoses, LECs need to be distinguished from dermoid and epidermoid cysts, which are also lined by squamous epithelium. The presence of mucinous cells and respiratory type mucosa favors a diagnosis of dermoid cysts [18]. The presence of splenic red pulp is diagnostic of epidermoid cysts [19]. The presence of hair follicles and respiratory mucosa in cysts lined by squamous epithelium with dense subepithelial lymphoid tissue of the nonsplenic type favors a diagnosis of LEC [2]. In our patient, the cystic wall was lined by stratified squamous epithelium and several lymphoid follicles and a few sebaceous glands were identified in the cystic wall. Although sebaceous glands are rare in LECs, a few authors have reported patients with LECs showing sebaceous glands [1,[9], [10], [11], [12]]. We could successfully diagnose a pancreatic LEC in our patient.

## 4 Conclusions

We report a rare case of a pancreatic LEC, which was difficult to be diagnosed preoperatively and summarized the imaging features of pancreatic cysts to differentiate from the malignancy.

## Conflicts of interest

The authors declare that they have no conflicts of interest.

## Sources of funding

No source of funding to be declared.

## Ethical approval

Ethical approval was not required and patient identifying knowledge was not presented in the report.

## Consent

Written informed consent was obtained from the patient for publication of this case report and accompanying images.

## Author contribution

Yosuke Namba: Drafted the manuscript.

Akihiko Oshita: Drafted the manuscript, supervised the writing of the manuscript and managed the patient.

Takashi Nishisaka: Diagnosed the pathological findings.

Tamito Sasaki and Maiko Namba: Managed the patient and contributed for the acquisition of data.

Yasuhiro Matsugu: Managed the patient

Toshiyuki Itamoto: Approved the final manuscript

## Registration of research studies

Our study does not require registration.

## Guarantor

Akihiko Oshita.

## Provenance and peer review

Not commissioned, externally peer-reviewed

## References

[1] Luchtrath H., Schriefers K.H. (1985). A pancreatic cyst with features of a so-called branchiogenic cyst. Pathologe.

[2] Adsay N.V., Hasteh F., Cheng J.D., Bejarano P.A., Lauwers G.Y., Batts K.P. (2002). Lymphoepithelial cysts of the pancreas: a report of 12 cases and a review of the literature. Mod. Pathol..

[3] Mege D., Gregoire E., Barbier L., Del Grande J., Le Treut Y.P. (2014). Lymphoepithelial cyst of the pancreas: an analysis of 117 patients. Pancreas.

[4] Kim W.H., Lee J.Y., Park H.S., Won H.J., Kim Y.H., Choi J.Y. (2013). Lymphoepithelial cyst of the pancreas: comparison of ct findings with other pancreatic cystic lesions. Abdom. Imaging.

[5] Agha R.A., Fowler A.J., Saetta A., Barai I., Rajmohan S., Orgill D.P., for the SCARE group (2016). The SCARE statement: consensus-based surgical case report guidelines. Int. J. Surg..

[6] Truong L.D., Rangdaeng S., Jordan P.H. (1987). Lymphoepithelial cyst of the pancreas. Am. J. Surg. Pathol..

[7] Ryu D.H., Sung R.H., Kang M.H., Choi J.W. (2015). Lymphoepithelial cyst of the pancreas mimicking malignant cystic tumor: report of a case. Korean J. Hepatobiliary Surg..

[8] Brugge W.R., Lauwers G.Y., Sahani D., Fernandez-del Castillo C., Warshaw A.L. (2004). Cystic neoplasms of the pancreas. New Engl. J. Med..

[9] Adsay N.V., Klimstra D.S., Compton C.C. (2000). Cystic lesions of the pancreas. Introduction Semin. Diagn. Pathol..

[10] Sewkani A., Purohit D., Singh V., Jain A., Varshney R., Varshney S. (2010). Lymphoepithelial cyst of the pancreas: a rare case report and review of literature. Indian J. Surg..

[11] Osiro S., Rodriguez J.R., Tiwari K.J., Rodriguez I.I., Mathenge N., Tubbs R.S. (2013). Is preoperative diagnosis possible? A clinical and radiological review of lymphoepithelial cysts of the pancreas. Jop.

[12] Lewandrowski K.B., Southern J.F., Pins M.R., Compton C.C., Warshaw A.L. (1993). Cyst fluid analysis in the differential diagnosis of pancreatic cysts. A comparison of pseudocysts, serous cystadenomas, mucinous cystic neoplasms, and mucinous cystadenocarcinoma. Ann. Surg..

[13] Yang J.M., Southern J.F., Warshaw A.L., Lewandrowski K.B. (1996). Proliferation tissue polypeptide antigen distinguishes malignant mucinous cystadenocarcinomas from benign cystic tumors and pseudocysts. Am. J. Surg..

[14] Domen H., Ohara M., Kimura N., Takahashi M., Yamabuki T., Komuro K. (2012). Lymphoepithelial cyst of the pancreas. Case Rep. Gastroenterol..

[15] Shinmura R., Gabata T., Matsui O. (2006). Lymphoepithelial cyst of the pancreas: case report with special reference to imaging--pathologic correlation. Abdom. Imaging.

[16] Nam S.J., Hwang H.K., Kim H., Yu JS Yoon D.S., Chung J.J. (2010). Lymphoepithelial cysts in the pancreas: Mri of two cases with emphasis of diffusion-weighted imaging characteristics. J. Magn. Reson. Imaging.

[17] Foley K.G., Christian A., Roberts S.A. (2012). Eus-fna diagnosis of a pancreatic lymphoepithelial cyst: three-year imaging follow-up. Jop..

[18] Kraimps J.L., Zins J., Levillain P., Azais O., Deleplanque G., Carretier M. (1993). Dermoid cyst of the pancreas. Eur. J. Surg..

[19] Adsay N.V., Hasteh F., Cheng J.D., Klimstra D.S. (2000). Squamous-lined cysts of the pancreas:Lymphoepithelial cysts, dermoid cysts (teratomas), and accessory-splenic epidermoid cysts. Semin. Diagn. Pathol..

